# Antibodies and Protection Against Invasive *Salmonella* Disease

**DOI:** 10.3389/fimmu.2014.00635

**Published:** 2014-12-22

**Authors:** Calman A. MacLennan

**Affiliations:** ^1^Novartis Vaccines Institute for Global Health, Siena, Italy; ^2^School of Immunity and Infection, College of Medicine and Dental Sciences, University of Birmingham, Birmingham, UK

**Keywords:** *Salmonella*, antibody, nontyphoidal, vaccine, invasive

Invasive disease caused by *Salmonella enterica* is a major global public health concern. It has two main clinical forms: enteric fever and invasive nontyphoidal *Salmonella* (iNTS) disease. Enteric fever imposes its highest burden of disease in South and South-East Asia and is principally caused by *S*. Typhi and *S*. Paratyphi A. Conversely, iNTS disease is a particular problem in sub-Saharan Africa where it is a leading cause of bacteremia (1, 2) and is mainly caused by *S*. Typhimurium and *S*. Enteritidis. As facultative intracellular bacteria, *Salmonellae* persist and multiply within the intracellular niche in macrophages but they are also capable of independent cell-free existence and this enables the spread of the infection from macrophage to macrophage. The way the immune system protects against these two phases of infection differs and this is key for developing a strategy to induce protective immunity against *Salmonella*. Antibodies have an important role in eliminating extracellular bacteria, while specific T cells are important for the clearance of intracellular bacteria. The contribution of these two arms of acquired immunity against *Salmonella* infections to protection has been an area of controversy in the past, and their relative importance is only now emerging. This opinion piece focuses on the role of antibodies in protecting against invasive *Salmonella* disease, and the application of this to vaccine development.

Epidemiological investigation, *in vitro* studies, animal models and vaccine studies indicate that antibodies can kill *Salmonella* that are not shielded by residing inside host cells. Conceptually, *Salmonellae* are exposed and therefore vulnerable to antibodies at distinct points of the invasion cycle: following initial invasion, when first entering the circulation, and when transiting from one phagocyte to another via the blood or extracellular fluids (3). An important consideration is the time that these bacteria are exposed to antibodies and whether this is sufficient for antibody-induced killing to occur. *In vitro* kinetic studies indicate that there is a window of opportunity of approximately 10 min before extracellular *S*. Typhimurium are killed by antibody and complement, and this time is sufficient to allow a proportion of bacteria entering the blood to escape into the intracellular niche (4).

Candidate vaccine studies in mice, where immunization is followed by challenge with live *Salmonella*, indicate the importance of antibodies for protection. Several studies have investigated experimental conjugate vaccines based on purified O polysaccharide from *Salmonella* (O-antigen; O:4,5 for *S*. Typhimurium and O:9 for *S*. Enteritidis) (5). Unlike intact lipopolysaccharides, these O-antigens lack lipid A and so are unable to act as thymus-independent type 1 (TI-1) antigens. Because of their repeating structure, they are likely to behave as thymus-independent type 2 (TI-2) antigens (6, 7) and therefore be capable of inducing *Salmonella*-specific antibodies, but not T cells. If O-antigen is associated with *Salmonella* protein, as it is in the intact bacterium or when present in membrane-vesicle-preparations, it has the potential to induce T-dependent B-cell immunity. T-cell help permits an immune response to the O-antigen in infants, affinity maturation of the antibody response and results in more persistent antibody production and the induction of memory.

Passive transfer studies of antibody from immune to non-immune animals have confirmed an important role for antibodies in protecting against *Salmonella* in mice. Nevertheless, the protection that antibody confers in this model depends on the inherent resistance to *Salmonella* of the mouse strain used, the virulence of the *Salmonella* strain, and the design of the challenge study. Optimal protection against *Salmonella* in mice requires a combination of antibodies and T cells. T cells appear to be most important for the late clearance of *Salmonella* infection (8), involving killing of intracellular bacteria from the macrophage beds of the spleen and liver.

There are a several drawbacks to studying *Salmonella* infections in mice as a model of disease in humans. These include the human restriction of *S*. Typhi and Paratyphi A, which limits mouse studies to nontyphoidal serovars. Also, there are differences in antibody-mediated immunity to *Salmonella* in mice and men. In man, antibodies can kill through direct complement-fixation and opsonophagocytosis, while in mice there appears to be little complement-mediated killing (9), leaving opsonophagocytic mechanisms to effect killing. In man, although there is evidence regarding the mechanisms of immune protection from vaccines against typhoid fever, no vaccine against NTS has progressed beyond a phase I clinical study. Hence, inferences regarding the mechanisms of immunity to iNTS disease in man come primarily from immunoepidemiological studies.

Of the two widely used types of vaccine against typhoid, Vi capsular polysaccharide (Vi CPS) vaccine, appears to operate entirely through the induction of protective antibody (5, 10). Similar to pure O-antigen, Vi polysaccharide is likely to be a TI-2 antigen. Despite lack of conjugation to a protein moiety, and hence lack of induction of T-cell immunity, the antibodies induced confer 55% 3-year protection (10, 11). New vaccines, where Vi CPS is conjugated to carrier proteins, such as tetanus toxoid, have been licensed recently for in-country use in India and China. These vaccines should provide greater protection than their unconjugated predecessor, albeit through more persistent and higher affinity Vi antibody production, rather than eliciting *Salmonella*-specific T cells, as the carrier proteins are usually not *Salmonella*-derived. The other widely used vaccine against typhoid is Ty21a, a live attenuated vaccine capable of inducing T cells as well as antibodies against *Salmonella*. Ty21a has a similar reported (51%) 3-year efficacy against typhoid as Vi CPS vaccine. Although Ty21a induces antibodies, none are directed against Vi, since it lacks Vi expression. The mechanisms by which Ty21a confers its protection are not well understood, but may include antibodies against the O:9 antigen which *S*. Typhi shares with *S*. Enteritidis.

The absence of a licensed vaccine against NTS means there is neither vaccine efficacy data nor a correlate of protection for iNTS disease. Consequently, evidence of the importance of antibodies in protection against iNTS disease relies on epidemiological evidence, which shows a correlation between fatal systemic iNTS disease and the period in childhood when naturally acquired antibodies are absent. This occurs after maternally-transmitted antibody has waned and before antibody has been induced through exposure, with peak incidence around one year of age (12). Although typhoid fever and iNTS disease are caused by bacteria belonging to the same species, extrapolating mechanisms of protection from the one disease to the other is not straightforward. One reason for this is the Vi capsule of *S*. Typhi which is absent from almost all nontyphoidal serovars of *Salmonella*. The capsule has immunomodulatory effects and has been shown to reduce inflammation (13–15). Additionally, it is now clear through genotyping that although the *S*. Typhimurium in sub-Saharan Africa is serologically indistinguishable from strains in the US and Europe, they belong to a different clade (16, 17).

Typhoid fever and iNTS disease have very different clinical manifestations and may require different approaches in order to effect protection by vaccination. Differences in their associated comorbidities, in particular, imply that the mechanisms of immune protection against these two forms of invasive *Salmonella* disease are not the same. HIV-infected individuals are highly susceptible to iNTS disease, while this association is not present with typhoid fever. Epidemiological data from Tanzania suggest a protective effect of HIV infection against typhoid, while an association between malaria and iNTS disease has long been recognized. Once again, no such link appears to exist with typhoid. Finally, individuals with deficiencies of the IL12/23-IFNγ cytokine axis (T_H_1 deficiencies) commonly present with iNTS disease, but not typhoid fever.

As discussed above, the acquisition of antibodies against NTS with age among African children corresponds to a fall in the incidence of episodes of iNTS disease (12), thus supporting a role for antibodies in protection against iNTS disease among young children. These antibodies have been shown to induce killing of *Salmonella* by phagocytes (18) and complement alone (12). More recently, this early acquisition of antibody-mediated immunity has been shown to correlate with levels of antibodies to O-antigen (19), supporting the development of a vaccine that induces such antibodies in order to protect young children in Africa against iNTS disease. Surprisingly, acquisition of *Salmonella*-specific T cells coincides with a peak in age-related iNTS disease incidence in African children (19), but these T cells could still play a secondary role in immunity to *Salmonella* among such children. Hence, in otherwise immune-competent children, a vaccine that can induce antibodies, particularly antibodies to O-antigen, appears likely to protect against iNTS disease. The early acquisition of antibodies to O-antigen occurs even in locations such as USA (20), where iNTS disease is uncommon, suggesting either ubiquitous sub-clinical exposure to NTS or the development of cross-reactive antibodies from other immune stimuli.

The reasons why HIV-infected individuals are susceptible to iNTS are more complex. While those with CD4 counts below 200/μl are most susceptible, the relevant mechanisms are probably more than just a reduction in CD4^+^ T cell-afforded protection. Dysregulation of anti-iNTS antibody-specific antibody production and cytokine responses, and increased invasion of *Salmonella* across the gastrointestinal wall also seem to be contributory, as does CD17^+^ T cell deficiency (2). Levels of antibodies against *Salmonella* O-antigen are much higher in some HIV-infected, compared with HIV-uninfected individuals and are associated with a lack of complement-mediated killing of *Salmonella in vitro* (21). Although the clinical significance of these findings is not entirely clear, we have recently described a similar occurrence in a group of patients with bronchiectasis and chronic *Pseudomonas aeruginosa* lung infection. High levels of IgG2 antibodies to the O-antigen of *Pseudomonas* are associated with both impaired *in vitro* killing of these bacteria, increased severity of respiratory infections and poor lung function (22). Nevertheless, anti-O-antigen antibodies are bactericidal at lower concentrations (23). Recurrent episodes of iNTS disease are a common problem among HIV-infected African adults. Many are caused by the same isolate of NTS suggesting persistence of *Salmonella* infection (24), even after clinical remission. With likely persistence in the intracellular niche and the importance of T cells for clearance of intracellular infection, a vaccine capable of inducing *Salmonella*-specific T cells may be more important in the context of HIV infection than the immune naivety of infancy.

Perhaps surprisingly, iNTS disease is not a common feature in individuals with primary antibody deficiencies, such as X-linked agammaglobulinemia or common variable immunodeficiency, although *Salmonella* gastrointestinal disease has been reported to be a problem in cohort studies of such patients (25). If, as other evidence indicates, antibody is key for protection against *Salmonella*, a lack of iNTS disease may be due to the low prevalence of *Salmonella* infections and absence of the ST313 invasive *S*. Typhimurium pathovar in industrialized settings, where it is possible to make a diagnosis of antibody deficiency. Alternatively, there may be redundancy in immunity to *Salmonella* by the time antibody levels wane and patients with CVID present with recurrent infections.

On the other hand, the high incidence of NTS granulomata among individuals with IL12/23-IFNγ cytokine axis deficiencies shows that antibodies (and complement) are insufficient alone to protect against iNTS disease in man. One series reported *Salmonella* disease in 43% of individuals with IL12/23 or IL12/23-receptor deficiencies (26). This cytokine axis is important for macrophage activation and elimination of intracellular bacteria, in particular *Salmonella* and mycobacteria. When tested, these patients have antibodies against *Salmonella*. Since they do not succumb to their *Salmonella* infections, it is plausible that, while not being sufficient to clear the macrophage beds of intracellular *Salmonella*, antibodies prevent fatal disease. As with HIV infection, the *Salmonella* serovars isolated are almost always nontyphoidal. Since IL12/23-IFNγ cytokine axis deficiencies affect signaling in response to *Salmonella* by innate/innate-like lymphocytes, including NK cells and γδ-T cells (27), the occurrence of *Salmonella* disease does not necessarily imply the need for *Salmonella*-specific T cells. iNTS disease is common among individuals with chronic granulomatous disease (25) and African children with malaria (28). In both cases, the phagocyte oxidative burst mechanism is impaired, suggesting the requirement for a functioning innate immune system to protect against iNTS disease.

In conclusion, vaccine efficacy studies strongly support a role for antibodies in protection against typhoid fever. Immunoepidemiological studies from Africa also support the importance of antibody for protection against fatal iNTS disease. However, strong clinical associations with secondary immunodeficiency due to HIV infection and malaria, and primary immunodeficiencies of the IL12/23-IFNγ cytokine axis and chronic granulomatous disease, suggest that antibody-mediated immunity against iNTS disease is only fully effective in the presence of an otherwise intact immune system. These observations indicate that bacteria from the same species (*Salmonella enterica*) not only cause different diseases, but that different immune mechanisms protect against these diseases. This conclusion may be applicable to other bacterial pathogens. While an antibody-inducing vaccine against iNTS disease may protect immunologically naive, but otherwise immunocompetent young children, it may be insufficient to protect individuals with primary and secondary immune deficiencies. Ultimately, efficacy studies with vaccines against NTS will be required to understand the importance of antibodies against iNTS disease. With no such vaccine currently even in early stage clinical trials, we are set for a long wait.

## Conflict of Interest Statement

Calman A. MacLennan is an employee of the Novartis Vaccines Institute for Global Health and recipient of a clinical research fellowship from GlaxoSmithKline.
